# Echocardiographic Assessment of Cardiovascular Hemodynamic Changes in Hypertensive Disorders of Pregnancy

**DOI:** 10.7759/cureus.104839

**Published:** 2026-03-07

**Authors:** Bharathi Venkatesh, Nikhitha Basavaraj, Ravindra S Pukale

**Affiliations:** 1 Obstetrics and Gynaecology, Adichunchanagiri Institute of Medical Sciences, Adichunchanagiri University, Mandya, IND

**Keywords:** 2d echo, maternal health, maternal outcome, obstetrics, obstetrics and gynecology residency, perinatal outcome, perinatal outcomes, pregnancy, pregnancy and heart disease, risk factors

## Abstract

Introduction

Hypertensive disorders of pregnancy are a major cause of maternal and perinatal morbidity and mortality worldwide, complicating a significant proportion of pregnancies. Cardiovascular maladaptation plays a central role in disease pathophysiology, yet routine echocardiographic evaluation is not universally practiced. The present study was conducted to evaluate cardiovascular hemodynamic alterations using two-dimensional echocardiography in women with hypertensive disorders of pregnancy and to compare echocardiographic parameters across different severity groups.

Materials and methods

This hospital-based prospective observational study included 70 pregnant women beyond 20 weeks of gestation with hypertensive disorders of pregnancy. Participants were stratified for analytical comparison into mild (19 (27.1%)), moderate (28 (40.0%)), and severe (23 (32.9%)) groups based on systolic and diastolic blood pressure levels. All women underwent a comprehensive echocardiographic assessment, including systolic parameters (left ventricular volumes, cardiac output, total vascular resistance) and diastolic parameters (E/A ratio, deceleration time, isovolumic relaxation time (IVRT)). Continuous variables were analyzed using one-way ANOVA, and categorical variables using the chi-square test. Pearson correlation assessed associations between blood pressure and echocardiographic parameters.

Results

Progressive increases in left ventricular end-systolic and end-diastolic volumes and cardiac output were observed with increasing disease severity (p < 0.001). Total vascular resistance decreased significantly across severity groups (p < 0.001). Diastolic dysfunction was present in 51 (72.9%) women and was more frequent in severe disease. The E/A ratio declined significantly with severity (p < 0.001). Significant correlations were noted between systolic blood pressure and cardiac output (r = 0.648, p < 0.001), and a significant negative correlation was observed with the E/A ratio (r = -0.612, p < 0.001). Severe disease was associated with higher rates of preterm delivery (17 (73.9%)), low birth weight (19 (82.6%)), and neonatal intensive care unit (NICU) admission (17 (73.9%)).

Conclusion

Hypertensive disorders of pregnancy are associated with significant systolic and diastolic hemodynamic alterations that correlate with disease severity and adverse maternal and neonatal outcomes. Two-dimensional echocardiographic evaluation provides valuable insight into cardiovascular involvement in hypertensive pregnancy; however, the findings should be interpreted in the context of an observational study without predictive modeling. However, larger studies with appropriate comparator groups are required to further clarify its clinical and prognostic significance.

## Introduction

Hypertensive disorders of pregnancy (HDP) remain a major global public health concern and are among the leading causes of maternal and perinatal morbidity and mortality worldwide [1]. These disorders complicate approximately 5-10% of all pregnancies, with variations across geographical regions and healthcare systems [2]. In India, the burden is substantial, with hypertension in pregnancy affecting nearly 7.8% of pregnant women and contributing to 10-15% of maternal deaths, particularly in resource-constrained settings [3]. The significant maternal and neonatal risks associated with HDP underscore the importance of understanding the cardiovascular alterations associated with disease severity and systemic involvement [1].

The spectrum of HDP includes gestational hypertension, pre-eclampsia, eclampsia, chronic hypertension, and chronic hypertension with superimposed pre-eclampsia [4]. Among these, pre-eclampsia represents the most clinically challenging entity due to its unpredictable progression and potential for multisystem involvement. The underlying pathophysiology involves abnormal placentation, endothelial dysfunction, systemic inflammation, and maladaptive cardiovascular responses. These alterations disrupt the normal physiological adaptations of pregnancy and predispose affected women to significant hemodynamic instability [5].

Normal pregnancy is characterized by marked cardiovascular adaptations, including increased plasma volume, elevated cardiac output, reduced systemic vascular resistance, and remodeling of cardiac chambers to accommodate increased circulatory demands [6]. In contrast, HDP are associated with impaired cardiovascular adaptation, leading to increased afterload, altered ventricular geometry, and functional abnormalities [2]. Studies have demonstrated that women with pre-eclampsia exhibit more pronounced structural and functional cardiac changes compared to those with uncomplicated gestational hypertension, particularly in severe disease [7]. Echocardiographic assessments have shown increased left ventricular mass, concentric remodeling, and significant diastolic dysfunction, with occasional systolic impairment in advanced cases. Importantly, these cardiovascular changes may persist beyond pregnancy and contribute to long-term cardiovascular risk [8].

Two-dimensional echocardiography is a safe, non-invasive, and widely available imaging modality that allows comprehensive assessment of cardiac structure and function during pregnancy without exposing the fetus to ionizing radiation [9]. It enables evaluation of ventricular volumes, cardiac output, vascular resistance, and diastolic filling patterns, providing valuable insight into hemodynamic alterations associated with HDP [10]. Despite its clinical utility, routine echocardiographic assessment is not consistently integrated into the evaluation of hypertensive pregnant women in many settings, and data regarding the pattern of cardiovascular involvement in Indian women remain limited.

Furthermore, although several studies have explored cardiovascular alterations in pre-eclampsia, most investigations focus on isolated echocardiographic parameters or on severe disease alone. Comprehensive evaluation of both systolic and diastolic hemodynamic changes across the spectrum of HDP remains limited, particularly in the Indian population, where demographic and healthcare factors may influence disease presentation. This lack of region-specific data represents an important gap in the literature and highlights the need for studies evaluating detailed cardiovascular hemodynamic changes in women with HDP.

Therefore, the present study aimed to observe cardiovascular hemodynamic alterations using two-dimensional echocardiography in women with HDP.

## Materials and methods

Study design and setting

This hospital-based prospective observational study was conducted in the Department of Obstetrics and Gynecology at Adichunchanagiri Institute of Medical Sciences (AIMS), B.G. Nagara, Karnataka, India, from June 2024 to November 2024. The study protocol was approved by the Institutional Ethics Committee (IEC No: AIMS/IEC/207/2024). Written informed consent was obtained from all participants prior to enrollment. The study was conducted in accordance with the Declaration of Helsinki and Good Clinical Practice guidelines.

Study population

Pregnant women beyond 20 weeks of gestation presenting with HDP were included in the study. Women were eligible if they had elevated blood pressure ≥140/90 mmHg documented after 20 weeks of gestation in previously normotensive women or had known chronic hypertension, consistent with ACOG definitions of HDP [11]. Singleton pregnancy was confirmed by first- or second-trimester ultrasonography. Eligible participants were between 18 and 40 years of age and provided written informed consent.

Women were excluded if they had known structural heart disease, valvular abnormalities, cardiomyopathy, or congenital heart disease. Additional exclusion criteria included multiple gestation, chronic kidney disease, pregestational diabetes mellitus, thyroid disorders, autoimmune diseases, chronic respiratory disease, intrauterine fetal demise, major fetal anomaly at enrolment, or poor echocardiographic window precluding adequate image acquisition.

Sample size estimation

The sample size was calculated based on the reported prevalence of Grade I diastolic dysfunction in HDP. In a Doppler echocardiographic study by Muthyala et al., Grade I diastolic dysfunction was observed in approximately 40% of women with preeclampsia [12]. Assuming a prevalence (P) of 40%, a 95% confidence level (Z = 1.96), and an absolute precision (d) of 11.5%, the required sample size was calculated using the formula,

$n = \frac{Z^{2} \times P(1 - P)}{d^{2}}$

After substituting the values minimum required sample size was 69.7. This was rounded to 70 participants, and consecutive eligible women were enrolled until the target sample size was achieved.

Clinical assessment

All enrolled participants underwent a comprehensive clinical evaluation, including detailed obstetric and medical history, physical examination, and blood pressure assessment. Blood pressure was measured in the left lateral recumbent position after five minutes of rest using a calibrated mercury sphygmomanometer with an appropriately sized cuff. Two readings were recorded and averaged. Mean arterial pressure (MAP) was calculated using the formula MAP = (SBP + 2 × DBP) / 3.

For analytical comparison, the severity of hypertensive disorder was stratified into three groups (mild, moderate, and severe) based on systolic and diastolic blood pressure ranges. Mild disease was defined as systolic blood pressure 140-159 mmHg and/or diastolic blood pressure 90-109 mmHg. Moderate disease was defined as systolic blood pressure 160-179 mmHg and/or diastolic blood pressure 110-119 mmHg. Severe disease was defined as systolic blood pressure ≥180 mmHg and/or diastolic blood pressure ≥120 mmHg or the presence of severe clinical features [2].

Baseline laboratory investigations included complete blood count, renal function tests, liver function tests, serum electrolytes, and urine albumin quantification. Obstetric ultrasonography was performed for fetal biometry and Doppler assessment, where indicated.

Echocardiographic assessment

Two-dimensional transthoracic echocardiography with color Doppler was performed within 24 hours of enrollment using a Philips EPIQ 7 ultrasound system equipped with a 2.5 MHz transducer. All examinations were conducted by experienced cardiologists following standardized protocols recommended by the American Society of Echocardiography (ASE) guidelines [13,14]. Participants were examined in the left lateral decubitus position, and measurements were averaged over three consecutive cardiac cycles.

Left ventricular end-systolic volume (LV ESV) and end-diastolic volume (LV EDV) were measured using the modified Simpson’s biplane method from apical four- and two-chamber views. Stroke volume was calculated as LV EDV minus LV ESV, and cardiac output was calculated as stroke volume multiplied by heart rate. Aortic root diameter and left ventricular outflow tract (LVOT) diameter were measured in the parasternal long-axis view at end-diastole. Total vascular resistance (TVR) was calculated using the formula TVR = (MAP / CO) × 80 and expressed in dyne/s/cm⁻⁵.

Diastolic function was assessed using pulsed-wave Doppler at the mitral valve leaflet tips. Parameters measured included E wave velocity, A wave velocity, E/A ratio, E wave deceleration time, isovolumic relaxation time (IVRT), and velocity-time integrals of E and A waves. Diastolic dysfunction was graded based on established Doppler criteria [13,14]. Grade I (impaired relaxation) was defined as an E/A ratio <1.0 with prolonged deceleration time and IVRT. Grade II (pseudonormal pattern) was defined as an E/A ratio of 1.0-1.5 with normal deceleration time. Grade III (restrictive pattern) was defined as an E/A ratio >2.0 with shortened deceleration time.

Outcome measures

Maternal outcomes assessed included mode of delivery and maternal intensive care unit admission. Neonatal outcomes included preterm delivery, birth weight less than 2500 grams, and neonatal intensive care unit (NICU) admission.

Statistical analysis

Statistical analysis was performed using IBM SPSS Statistics version 26.0 (IBM Corp., Armonk, NY). Continuous variables were expressed as mean ± standard deviation, and categorical variables were presented as N (%). Comparisons of continuous variables across severity groups were performed using one-way analysis of variance (ANOVA), and F statistics were reported. Categorical variables were compared using the chi-square test, and χ² statistics were reported. Pearson’s correlation coefficient was used to assess linear associations between blood pressure parameters and echocardiographic variables, and corresponding correlation coefficients (r) and p-values were reported. All enrolled participants had complete clinical and echocardiographic datasets; therefore, no missing data were present in the final analysis. A p-value <0.05 was considered statistically significant.

## Results

The study included 70 pregnant women with HDP. The majority were aged 20-25 years (30 (42.9%)), followed by 26-30 years (26 (37.1%)), with a mean age of 26.8 ± 4.2 years. Most participants were homemakers (50 (71.4%)) and resided in rural areas (45 (64.3%)). Multigravida women constituted 39 (55.7%), while 31 (44.3%) were primigravida. The mean gestational age was 34.2 ± 3.6 weeks, and the mean BMI was 26.4 ± 3.8 kg/m². The mean systolic and diastolic blood pressures were 156.8 ± 18.4 mmHg and 102.6 ± 12.8 mmHg, respectively, with a MAP of 120.7 ± 13.2 mmHg. The mean birth weight was 2468 ± 584 g (Table 1).

**Table 1:** Baseline Demographic and Clinical Characteristics (N = 70) This table summarizes the baseline demographic, obstetric, and clinical characteristics of the study participants diagnosed with hypertensive disorders of pregnancy (HDP). Categorical variables are presented as numbers and percentages (N (%)), and continuous variables are expressed as mean ± standard deviation (SD). The majority of participants were aged 20-30 years, predominantly homemakers, and from rural areas. More than half were multigravida. The mean gestational age was 34.2 weeks, and the mean BMI was 26.4 kg/m². Elevated mean systolic and diastolic blood pressures reflect the hypertensive status of the cohort. Neonatal birth weight is presented as mean ± SD.

Variable		N (%)/mean ± SD
Age (years)	20-25 years	30 (42.9%)
	26-30 years	26 (37.1%)
	31-35 years	12 (17.1%)
	>35 years	2 (2.9%)
	Mean ± SD	26.8 ± 4.2
Occupation	Homemaker	50 (71.4%)
	Employed	20 (28.6%)
Residence	Rural	45 (64.3%)
	Urban	25 (35.7%)
Socioeconomic status	Lower	25 (35.7%)
	Middle	37 (52.9%)
	Upper	8 (11.4%)
Parity	Primigravida	31 (44.3%)
	Multigravida	39 (55.7%)
Gestational age (weeks)	Mean ± SD	34.2 ± 3.6
BMI (kg/m²)	Mean ± SD	26.4 ± 3.8
Systolic BP (mmHg)	Mean ± SD	156.8 ± 18.4
Diastolic BP (mmHg)	Mean ± SD	102.6 ± 12.8
Mean arterial pressure (mmHg)	Mean ± SD	120.7 ± 13.2
Birth weight (g)	Mean ± SD	2468 ± 584

Regarding severity distribution, participants were stratified for analytical comparison into mild, moderate, and severe hypertensive disorder groups based on blood pressure ranges. A total of 19 (27.1%) participants had mild disease, 28 (40.0%) had moderate disease, and 23 (32.9%) had severe hypertensive disorders with severe clinical features, indicating that nearly one-third of the cohort presented with severe disease (Table 2).

**Table 2:** Distribution According to Severity of Hypertensive Disorders of Pregnancy (N = 70) This table depicts the distribution of participants according to disease severity. Moderate hypertensive disorder was the most common presentation, followed by severe disease. Nearly one-third of the cohort presented with severe disease, highlighting a substantial burden of advanced hypertensive pathology in the study population. Data are expressed as numbers and percentages (N (%)).

Severity	N (%)
Mild	19 (27.1%)
Moderate	28 (40.0%)
Severe (with severe features)	23 (32.9%)

Significant progressive alterations in systolic cardiac parameters were observed with increasing disease severity. LV ESV increased from 32.4 ± 6.8 mL in mild cases to 42.6 ± 9.4 mL in severe cases (F = 11.82, p < 0.001), while left ventricular end-diastolic volume increased from 92.8 ± 10.4 mL to 106.2 ± 14.6 mL (F = 9.67, p < 0.001). Cardiac output also showed a significant rise across severity groups (4.8 ± 0.9 to 5.6 ± 1.1 L/min; F = 6.94, p < 0.001). Conversely, TVR demonstrated a significant decline from 1456 ± 312 to 1142 ± 268 dyne/s/cm⁵ (F = 10.43, p < 0.001). Stroke volume and LVOT diameter did not differ significantly (p > 0.05) (Table 3).

**Table 3:** Comparison of Echocardiographic Systolic Parameters Across Severity Groups This table compares echocardiographic systolic function parameters among mild, moderate, and severe hypertensive disorder groups. Values are expressed as mean ± SD. Progressive increases in left ventricular end-systolic volume (LV ESV), left ventricular end-diastolic volume (LV EDV), cardiac output, and aortic root diameter were observed with increasing disease severity. Total vascular resistance showed a significant decline across severity categories. Stroke volume and left ventricular outflow tract (LVOT) diameter did not differ significantly. Statistical comparisons were performed using one-way ANOVA, with corresponding F values and p-values reported. LV EDV - left ventricular end-diastolic volume; LV ESV - left ventricular end-systolic volume; LVOT - left ventricular outflow tract

Parameter	Mild (n = 19) mean ± SD	Moderate (n = 28) mean ± SD	Severe (n = 23) mean ± SD	F-value	p-value
LV ESV (mL)	32.4 ± 6.8	36.2 ± 7.6	42.6 ± 9.4	11.82	<0.001
LV EDV (mL)	92.8 ± 10.4	98.4 ± 11.2	106.2 ± 14.6	9.67	<0.001
Stroke volume (mL)	60.4 ± 8.2	62.2 ± 9.4	63.6 ± 10.8	1.02	0.36
Cardiac output (L/min)	4.8 ± 0.9	5.2 ± 0.8	5.6 ± 1.1	6.94	<0.001
Aortic root diameter (cm)	2.86 ± 0.32	2.94 ± 0.36	3.08 ± 0.42	4.88	0.01
LVOT (cm)	2.02 ± 0.24	2.06 ± 0.26	2.12 ± 0.28	1.41	0.25
Total vascular resistance (dyne/s/cm⁵)	1456 ± 312	1298 ± 284	1142 ± 268	10.43	<0.001

Diastolic function parameters showed significant worsening with increasing severity. The E/A ratio decreased progressively from 1.12 ± 0.16 in mild cases to 0.89 ± 0.18 in severe cases (F = 14.27, p < 0.001). E-wave velocity decreased while A-wave velocity increased significantly (p < 0.05). E-wave deceleration time increased from 192 ± 25 ms to 239 ± 34 ms (F = 18.64, p < 0.001), and IVRT increased from 82 ± 12 ms to 108 ± 18 ms (F = 16.81, p < 0.001), indicating progressive impairment of left ventricular relaxation (Table 4).

**Table 4:** Comparison of Echocardiographic Diastolic Parameters Across Severity Groups This table presents the comparison of diastolic function parameters across severity groups. Data are expressed as mean ± SD and analyzed using one-way ANOVA. A progressive decline in E-wave velocity and E/A ratio, along with significant increases in A-wave velocity, E-wave deceleration time, and IVRT, were observed with increasing disease severity. These findings indicate worsening left ventricular relaxation and diastolic dysfunction in severe hypertensive disorders of pregnancy. IVRT - isovolumetric relaxation time; VTI - velocity time integral

Parameter	Mild mean ± SD	Moderate mean ± SD	Severe mean ± SD	F-value	p-value
E wave (cm/s)	84 ± 14	78 ± 16	72 ± 18	5.96	<0.001
A wave (cm/s)	75 ± 12	79 ± 14	81 ± 16	3.89	0.02
E/A ratio	1.12 ± 0.16	0.98 ± 0.14	0.89 ± 0.18	14.27	<0.001
E deceleration time (ms)	192 ± 25	217 ± 28	239 ± 34	18.64	<0.001
IVRT (ms)	82 ± 12	94 ± 16	108 ± 18	16.81	<0.001
E VTI (cm)	12.4 ± 2.2	11.2 ± 2.6	10.2 ± 2.8	6.02	0.004
A VTI (cm)	6.2 ± 1.4	6.8 ± 1.6	7.4 ± 1.8	5.33	0.007

Correlation analysis demonstrated significant associations between blood pressure parameters and echocardiographic variables. Systolic blood pressure showed strong positive correlations with LV end-systolic volume (r = 0.624, p < 0.001) and cardiac output (r = 0.648, p < 0.001), and a significant negative correlation with E/A ratio (r = -0.612, p < 0.001). Similar patterns were observed with diastolic blood pressure and MAP, all showing statistically significant correlations (p < 0.001), indicating that increasing blood pressure was associated with worsening systolic and diastolic dysfunction (Table 5).

**Table 5:** Correlation Between Blood Pressure Parameters and Echocardiographic Variables (N = 70) This table illustrates the correlation between blood pressure parameters (systolic blood pressure (SBP), diastolic blood pressure (DBP), and mean arterial pressure (MAP)) and selected echocardiographic variables. Continuous variables are presented as mean ± SD. Pearson’s correlation coefficient (r) and corresponding p-values are reported. Significant positive correlations were observed between blood pressure indices and LV volumes and cardiac output, while significant negative correlations were noted with E/A ratio and total vascular resistance. These findings demonstrate a strong association between increasing blood pressure levels and both systolic and diastolic cardiac dysfunction.

Echocardiographic Variable	Mean ± SD	SBP		DBP		MAP	
		r	p-value	r	p-value	r	p-value
LV ESV (mL)	37.6 ± 8.9	0.624	<0.001	0.652	<0.001	0.642	<0.001
LV EDV (mL)	99.8 ± 13.1	0.582	<0.001	0.608	<0.001	0.596	<0.001
Cardiac output (L/min)	5.2 ± 1.0	0.648	<0.001	0.672	<0.001	0.662	<0.001
E/A ratio	0.99 ± 0.19	-0.612	<0.001	-0.638	<0.001	-0.628	<0.001
IVRT (ms)	95 ± 18	0.542	<0.001	0.586	<0.001	0.568	<0.001
Total vascular resistance (dyne/s/cm⁵)	1298 ± 301	-0.594	<0.001	-0.618	<0.001	-0.608	<0.001

Assessment of diastolic dysfunction grading revealed that only 19 (27.1%) participants had normal diastolic function, while 51 (72.9%) exhibited some degree of dysfunction. Grade I dysfunction was the most common pattern across all groups, observed in 10 (52.6%) mild, 16 (57.1%) moderate, and 15 (65.2%) severe cases. Higher grades (Grades II and III) were more frequently seen in severe disease. The distribution differed significantly across severity groups (χ² = 9.42, p = 0.024) (Table 6).

**Table 6:** Distribution of Diastolic Dysfunction Across Severity Groups This table shows the distribution of diastolic dysfunction grades among mild, moderate, and severe hypertensive disorder groups. Data are presented as numbers and percentages (N (%)). Grade I diastolic dysfunction was the most common pattern across all severity categories, while higher grades (Grades II and III) were more frequently observed in severe disease. The distribution differed significantly across groups, as determined by the chi-square test (χ² = 9.42, p = 0.024), indicating a significant association between disease severity and worsening diastolic dysfunction.

Diastolic function	Mild (n = 19) N (%)	Moderate (n = 28) N (%)	Severe (n = 23) N (%)
Normal	7 (36.8%)	8 (28.6%)	4 (17.4%)
Grade I	10 (52.6%)	16 (57.1%)	15 (65.2%)
Grade II	2 (10.5%)	3 (10.7%)	3 (13.0%)
Grade III	0 (0%)	1 (3.6%)	1 (4.3%)
χ² value	9.42		
p-value	0.024		

Maternal and neonatal outcomes worsened significantly with increasing disease severity. Cesarean delivery rates increased from 10 (52.6%) in mild cases to 18 (78.3%) in severe cases (χ² = 6.18, p = 0.045). Preterm delivery was significantly higher in severe disease (17 (73.9%)) compared to mild disease (5 (26.3%)) (χ² = 12.74, p < 0.001). Low birth weight (<2500 g) was observed in 19 (82.6%) severe cases (χ² = 14.83, p < 0.001). NICU admission and maternal ICU admission were also significantly more frequent in severe disease (17 (73.9%) and six (26.1%), respectively; p ≤ 0.015), demonstrating a clear association between hypertensive severity and adverse outcomes (Table 7).

**Table 7:** Maternal and Neonatal Outcomes Across Severity Groups This table compares maternal and neonatal outcomes across severity groups of hypertensive disorders of pregnancy. Data are presented as numbers and percentages (N (%)) and analyzed using the chi-square test. Cesarean delivery rates, preterm birth, low birth weight (<2500 g), neonatal intensive care unit (NICU) admission, and maternal intensive care unit (ICU) admission increased significantly with disease severity. These findings demonstrate a clear and statistically significant association between increasing hypertensive severity and adverse maternal and neonatal outcomes.

Outcome	Mild N (%)	Moderate N (%)	Severe N (%)	χ² value	p-value
Cesarean delivery	10 (52.6%)	20 (71.4%)	18 (78.3%)	6.18	0.045
Preterm delivery	5 (26.3%)	13 (46.4%)	17 (73.9%)	12.74	<0.001
Birth weight <2500g	7 (36.8%)	15 (53.6%)	19 (82.6%)	14.83	<0.001
NICU admission	5 (26.3%)	10 (35.7%)	17 (73.9%)	13.02	<0.001
Maternal ICU admission	1 (5.3%)	3 (10.7%)	6 (26.1%)	8.41	0.015

## Discussion

The present study demonstrates significant and progressive cardiovascular hemodynamic alterations in pregnant women with hypertensive disorders, as evaluated by two-dimensional echocardiography. Nearly one-third of participants presented with severe disease (23 (32.9%)), reflecting the referral pattern to a tertiary care center. The majority were multigravida (39 (55.7%)), supporting the prior observation that hypertensive disorders affect both primigravida and multigravida women [15]. Participants were stratified into mild, moderate, and severe groups for analytical comparison, and the progressive changes in echocardiographic parameters across these groups highlight the relationship between hypertensive severity and cardiac structural and functional adaptation.

The present study findings of increasing left ventricular end-diastolic and end-systolic volumes with worsening disease severity are consistent with previous reports of ventricular remodeling in hypertensive pregnancy. Kametas et al. described similar increases in ventricular dimensions among women with pre-eclampsia, attributing these alterations to increased afterload and altered myocardial compliance [16]. In our cohort, LV ESV increased from 32.4 ± 6.8 mL in mild cases to 42.6 ± 9.4 mL in severe cases (p < 0.001), suggesting a graded structural response to sustained hypertensive stress. Cardiac output also demonstrated a progressive rise from 4.8 ± 0.9 L/min in mild disease to 5.6 ± 1.1 L/min in severe disease (p < 0.001), indicating a predominantly hyperdynamic circulatory pattern. This aligns with the concept of heterogeneous hemodynamic phenotypes in pre-eclampsia described by Vasapollo et al., where a subset of women exhibit elevated cardiac output with compensatory vascular changes [17].

An important observation in the present study was the significant decline in TVR with increasing disease severity, despite elevated blood pressure levels. This pattern has been described in selected cohorts and may represent a compensatory adaptation to maintain systemic perfusion in the presence of increased cardiac output [18]. This apparent paradox may reflect heterogeneous hemodynamic responses in hypertensive pregnancy, where increased cardiac output partially offsets elevated vascular tone. However, compared to normal pregnancy physiology, where systemic vascular resistance typically declines substantially, TVR in the severe group remained relatively elevated, reflecting persistent vascular dysfunction. These findings emphasize that HDP are not uniformly vasoconstrictive states but rather complex hemodynamic syndromes with variable phenotypic expression.

Diastolic dysfunction emerged as the predominant echocardiographic abnormality in the present study. Overall, 51 (72.9%) women exhibited some degree of diastolic dysfunction, with a higher prevalence in severe disease. The E/A ratio declined significantly from 1.12 ± 0.16 in mild cases to 0.89 ± 0.18 in severe cases (p < 0.001), accompanied by prolongation of IVRT and deceleration time, indicating impaired myocardial relaxation. These findings are consistent with the systematic review by Castleman et al. [19], which identified diastolic dysfunction as the most consistent cardiac abnormality in hypertensive pregnancy, often preceding overt systolic impairment. The underlying mechanisms likely involve increased afterload, concentric remodeling, myocardial stiffness, endothelial dysfunction, and inflammation-mediated alterations in myocardial relaxation [20].

The strong correlations between blood pressure parameters and echocardiographic indices further reinforce the cardiovascular impact of hypertensive stress. Positive correlations between systolic blood pressure and cardiac output (r = 0.648, p < 0.001), along with inverse correlations with E/A ratio (r = -0.612, p < 0.001), suggest that increasing blood pressure is directly associated with heightened cardiac workload and worsening diastolic function. Clinically, adverse maternal and neonatal outcomes were more frequent in severe disease, including preterm delivery (17 (73.9%)), low birth weight (19 (82.6%)), and NICU admission (17 (73.9%)), indicating that cardiac dysfunction parallels disease severity and perinatal risk. While these findings highlight the clinical relevance of cardiovascular alterations in hypertensive pregnancy, the observational design of the study limits conclusions regarding causality or predictive value. Given evidence that HDP confer increased long-term cardiovascular risk, including heart failure and ischemic heart disease, identification of echocardiographic abnormalities during pregnancy may provide additional descriptive insight into cardiovascular involvement, and further longitudinal studies are needed to determine their prognostic significance [21].

Strengths and limitations

The strengths of this study include its prospective design, standardized echocardiographic assessment, and comprehensive evaluation of both systolic and diastolic parameters across clearly defined severity groups, allowing correlation of hemodynamic changes with clinical outcomes. However, the study has certain limitations, including its single-center design and relatively modest sample size of 70 participants, which may limit generalizability. The absence of a normotensive control group restricts direct comparison with normal pregnancy-related cardiovascular adaptations. Additionally, the cross-sectional design without postpartum follow-up prevents assessment of the persistence or reversibility of observed cardiac abnormalities. Furthermore, multivariable regression or predictive modeling was not performed, and prognostic value or independent predictors of adverse outcomes were not identified.

## Conclusions

The findings of this study indicate that HDP are associated with measurable alterations in cardiovascular hemodynamics, involving both systolic and diastolic function, with greater abnormalities observed in more severe disease. The observed associations between blood pressure levels, echocardiographic parameters, and adverse maternal and neonatal outcomes suggest that cardiac involvement is closely related to the clinical severity of HDP. Two-dimensional echocardiography provides valuable insights into these hemodynamic changes and may contribute to improved understanding of cardiovascular involvement in affected pregnancies. However, in the absence of a normotensive comparator group and longitudinal follow-up, definitive conclusions regarding the extent of deviation from normal pregnancy physiology and long-term implications cannot be drawn from the present observational study. Future studies incorporating appropriate control groups, larger multicentric cohorts, and serial postpartum follow-up are warranted to better delineate the trajectory, reversibility, and prognostic significance of cardiac changes in HDP.

## References

[1] Zerihun E, Girma F, Amena N (2025). Effect of Hypertensive Disorders of Pregnancy (HDP) on maternal and perinatal birth outcomes in Eastern Ethiopia: a prospective cohort study. BMC Pregnancy Childbirth.

[2] Khedagi AM, Bello NA (2021). Hypertensive disorders of pregnancy. Cardiol Clin.

[3] Abalos E, Cuesta C, Grosso AL, Chou D, Say L (2013). Global and regional estimates of preeclampsia and eclampsia: a systematic review. Eur J Obstet Gynecol Reprod Biol.

[4] Booker WA (2020). Hypertensive disorders of pregnancy. Clin Perinatol.

[5] Chaiworapongsa T, Chaemsaithong P, Yeo L, Romero R (2014). Pre-eclampsia part 1: current understanding of its pathophysiology. Nat Rev Nephrol.

[6] Sanghavi M, Rutherford JD (2014). Cardiovascular physiology of pregnancy. Circulation.

[7] Shivananjiah C, Nayak A, Swarup A (2016). Echo changes in hypertensive disorder of pregnancy. J Cardiovasc Echogr.

[8] Melchiorre K, Sutherland GR, Liberati M, Thilaganathan B (2011). Preeclampsia is associated with persistent postpartum cardiovascular impairment. Hypertension.

[9] Cong J, Fan T, Yang X, Squires JW, Cheng G, Zhang L, Zhang Z (2015). Structural and functional changes in maternal left ventricle during pregnancy: a three-dimensional speckle-tracking echocardiography study. Cardiovasc Ultrasound.

[10] Chen ZH, Chiu WH, Chao SS, Deng L, Li L, Shikano R, Liu JB (2026). Echocardiographic investigation of maternal cardiac physiological adaptations in Chinese pregnancies. Sci Rep.

[11] (2020). Gestational hypertension and preeclampsia: ACOG Practice Bulletin, Number 222. Obstet Gynecol.

[12] Muthyala T, Mehrotra S, Sikka P, Suri V (2016). Maternal cardiac diastolic dysfunction by Doppler echocardiography in women with preeclampsia. J Clin Diagn Res.

[13] Lang RM, Badano LP, Mor-Avi V (2015). Recommendations for cardiac chamber quantification by echocardiography in adults: an update from the American Society of Echocardiography and the European Association of Cardiovascular Imaging. J Am Soc Echocardiogr.

[14] Nagueh SF, Smiseth OA, Appleton CP (2016). Recommendations for the evaluation of left ventricular diastolic function by echocardiography: an update from the American Society of Echocardiography and the European Association of Cardiovascular Imaging. J Am Soc Echocardiogr.

[15] Parikh PM, Goswami VM, Shah SR (2021). Significance of 2 dimensional echocardiography in hypertensive disorders of pregnancy: a study in tertiary care centre, Ahmedabad, Western India. Int J Reprod Contracept Obstet Gynecol.

[16] Kametas NA, McAuliffe F, Hancock J, Chambers J, Nicolaides KH (2001). Maternal left ventricular mass and diastolic function during pregnancy. Ultrasound Obstet Gynecol.

[17] Vasapollo B, Novelli GP, Valensise H (2008). Total vascular resistance and left ventricular morphology as screening tools for complications in pregnancy. Hypertension.

[18] Tihtonen K, Kööbi T, Huhtala H, Uotila J (2007). Hemodynamic adaptation during pregnancy in chronic hypertension. Hypertens Pregnancy.

[19] Castleman JS, Ganapathy R, Taki F, Lip GY, Steeds RP, Kotecha D (2016). Echocardiographic structure and function in hypertensive disorders of pregnancy: a systematic review. Circ Cardiovasc Imaging.

[20] Spaanderman ME, Meertens M, van Bussel M, Ekhart TH, Peeters LL (2000). Cardiac output increases independently of basal metabolic rate in early human pregnancy. Am J Physiol Heart Circ Physiol.

[21] Bellamy L, Casas JP, Hingorani AD, Williams DJ (2007). Pre-eclampsia and risk of cardiovascular disease and cancer in later life: systematic review and meta-analysis. BMJ.

